# Transcriptome analysis reveals differentially expressed genes associated with germ cell and gonad development in the Southern bluefin tuna (Thunnus maccoyii)

**DOI:** 10.1186/s12864-016-2397-8

**Published:** 2016-03-10

**Authors:** Ido Bar, Scott Cummins, Abigail Elizur

**Affiliations:** Genecology Research Centre, Faculty of Science, Health, Education and Engineering, University of the Sunshine Coast, 4558 Maroochydore DC, Queensland, Australia

**Keywords:** Southern bluefin tuna, RNA-Sequencing, Illumina, Spermatogonia, Reproduction, Germ cell transplantation

## Abstract

**Background:**

Controlling and managing the breeding of bluefin tuna (*Thunnus* spp.) in captivity is an imperative step towards obtaining a sustainable supply of these fish in aquaculture production systems. Germ cell transplantation (GCT) is an innovative technology for the production of inter-species surrogates, by transplanting undifferentiated germ cells derived from a donor species into larvae of a host species. The transplanted surrogates will then grow and mature to produce donor-derived seed, thus providing a simpler alternative to maintaining large-bodied broodstock such as the bluefin tuna. Implementation of GCT for new species requires the development of molecular tools to follow the fate of the transplanted germ cells. These tools are based on key reproductive and germ cell-specific genes. RNA-Sequencing (RNA-Seq) provides a rapid, cost-effective method for high throughput gene identification in non-model species. This study utilized RNA-Seq to identify key genes expressed in the gonads of Southern bluefin tuna (*Thunnus maccoyii*, SBT) and their specific expression patterns in male and female gonad cells.

**Results:**

Key genes involved in the reproductive molecular pathway and specifically, germ cell development in gonads, were identified using analysis of RNA-Seq transcriptomes of male and female SBT gonad cells. Expression profiles of transcripts from ovary and testis cells were compared, as well as testis germ cell-enriched fraction prepared with Percoll gradient, as used in GCT studies. Ovary cells demonstrated over-expression of genes related to stem cell maintenance, while in testis cells, transcripts encoding for reproduction-associated receptors, sex steroids and hormone synthesis and signaling genes were over-expressed. Within the testis cells, the Percoll-enriched fraction showed over-expression of genes that are related to post-meiosis germ cell populations.

**Conclusions:**

Gonad development and germ cell related genes were identified from SBT gonads and their expression patterns in ovary and testis cells were determined. These expression patterns correlate with the reproductive developmental stage of the sampled fish. The majority of the genes described in this study were sequenced for the first time in *T. maccoyii*. The wealth of SBT gonadal and germ cell-related gene sequences made publicly available by this study provides an extensive resource for further GCT and reproductive molecular biology studies of this commercially valuable fish.

**Electronic supplementary material:**

The online version of this article (doi:10.1186/s12864-016-2397-8) contains supplementary material, which is available to authorized users.

## Introduction

Bluefin tunas (*Thunnus* spp.) provide some of the highest valued fish in the fresh and frozen international fish market and as such, they are highly susceptible to over-fishing, resulting in strict fishing regulations and quotas, which limit the available catch [1]. In order to maintain a sustainable supply of bluefin tuna to meet the ever-growing demand, without seasonal or regional constrains, and relieve the fishing pressure from wild stocks, bluefin tuna supply should turn to aquaculture based production systems [2]. To achieve this goal, bluefin tuna broodstock must be bred in captivity and therefore extensive research has been invested into facilitating the reproduction in captivity and broodstock management of three major bluefin tuna species: Pacific bluefin tuna (PBT, *Thunnus orientalis*), Atlantic bluefin tuna (BFT, *Thunnus thynnus thynnus*) and Southern bluefin tuna (SBT, *Thunnus maccoyii*).

Molecular techniques and tools are becoming increasingly helpful to identify key pathways and biological processes involved in bluefin tunas reproductive biology. Such molecular research includes the discovery of the Kisspeptin system in SBT [3], treatment of gonadotropin releasing hormone agonist (Gnrha) to induce spawning in BFT and SBT [4, 5], description of gene expression patterns in gonads of BFT [6, 7] and identification of germ cell specific molecular markers in PBT [8]. Development of these molecular tools requires identification of genes and control elements coded in the DNA sequence and expressed as mRNA transcripts.

Germ cell transplantation (GCT) is an innovative molecular biotechnology for the production of inter-species surrogates, capable of facilitating easy handling, shorter generation time and a more economical management of large-bodied broodstock, such as the bluefin tuna. This technology involves the isolation of undifferentiated type-A spermatogonia (ASG) from a donor fish testis and transplanting these germ cell precursors into larvae of a host fish that will become a surrogate producing donor-derived gametes upon sexual maturation [9, 10]. The isolation of spermatogonia from the donor testis is achieved by mechanical and enzymatic dissociation of the testis, often followed by a second cell selection step using a discontinuous Percoll density gradient [11–13].

Implementation of GCT technology for a new species, however, requires development of molecular tools similar to those described above to identify transplantable germ cell populations in the donor gonads, to track the migration and settlement of the transplanted germ cells once in the surrogate host and to detect the presence of donor-derived DNA and RNA in the maturing hosts’ gonads and gametes [10, 14]. This approach was demonstrated previously in this study to distinguish between transplanted SBT germ cells and yellowtail kingfish (YTK, *Seriola lalandi*) hosts endogenous cells, using the 3’ untranslated region (UTR) of the germ cell-specific *vasa* gene. The transplanted SBT cells, however, did not proliferate and further differentiate in the YTK host, most likely due to molecular incompatibilities derived from the evolutionary distance between the two species [15]. Moreover, because the host species should preferably be as phylogenetically close to the donor species, a higher level of homology is expected between the genes of the two species, therefore a wide range of molecular markers is needed to ensure that some would be divergent enough to allow for species-specific identification.

Isolation of genes in non-model organisms, such as the bluefin tuna species, has typically relied on degenerate-primer polymerase chain reaction (PCR) amplification of candidate genes, followed by sequencing. This method, however, is time consuming as it needs to be performed for each individual gene with substantial trial-and-error to clone the gene of interest. Furthermore, it requires prior knowledge of conserved regions of the candidate genes in other species, preferably as phylogenetically close as possible to the target species. This requirement presents a major bottleneck for gene discovery for the SBT, because there is a lack of gene sequences available in the public databases: currently less than 300 combined nucleotide and protein sequences and only 13 annotated genes can be found for SBT (taxonomy ID: 8240) in the National Center for Biotechnology Information (NCBI) databases [16]. However, the recently published genome of the PBT [17], together with nucleotide and protein sequences from the entire *Thunnus* genus (taxonomy id: 8234) available on NCBI, provide good reference for comparative discovery of genes in the closely related SBT, to overcome the lack of publicly available sequence data.

Gene discovery methods are evolving rapidly from the traditional methods described above, to high throughput next generation sequencing (NGS), as a result of decreasing costs, fast processing times and a plethora of emerging analysis tools [18–21]. In a similar manner, gene expression data acquired with NGS RNA-Seq can cover the entire transcriptome of a sample in a single analysis, and serve as an alternative to individual gene real-time quantitative PCR and higher throughput microarrays, both of which require prior sequence knowledge [22].

This study aimed at identifying genes differentially expressed in male and female gonads of SBT. Special attention was given to genes known to be involved in germ cell differentiation and proliferation, to develop molecular tools for implementation of GCT for SBT. Specifically, markers for undifferentiated transplantable gonadal stem cells, ASG from testes and oogonia from ovaries, were sought after to enable their isolation and detection with molecular methods before and after transplantation. To achieve this, transcriptomes of SBT gonadal cells were assembled: crude cell extracts from ovary, testis, Percoll-enriched germ cells (as used in GCT experiments) and oogonia-enriched filtered cells. The transcriptomes were constructed using a combined approach of *de novo* and genome guided assembly of NGS RNA-Seq, with the PBT genome as a closely-related species reference. The transcriptomes were used to compare gene expression of SBT ovary and testis cells and evaluate currently practiced ASG and oogonia cell enrichment methods used to prepare germ cells for GCT. In addition, the discovery of reproduction-related genes and their expression profiles in male and female SBT gonads is described as a foundation for future use of molecular tools in SBT reproductive research towards successful broodstock management and spawning in captivity.

## Methods

### Sample collection

SBT gonads were collected during a commercial harvest at Cleanseas Tuna Ltd. sea-cages offshore Port Lincoln, South Australia. Twelve (12) fish were killed and immobilized by the harvest crew, then measured for weight (gilled and gutted), total length (TL) and examined for sex and gonad collection (details in Table 1). Similar sized fish were selected to try and minimize size and sexual development related variation between and within groups. The gonads were washed briefly with ice cold 10 mM phosphate buffered solution (PBS, P4117; Sigma-Aldrich) immediately after removal, transferred into 100 mL clean PBS and kept on ice during transport back to the laboratory, approximately 4 h after collection. Gonado-somatic index (GSI), presented as percentage, was calculated for each fish as the ratio between gonad weight to gilled and gutted weight (in grams).

**Table 1:** Details of Southern bluefin tuna sampled for RNA extraction

Fish	Fish weight^a^ (kg)	Gonad weight (g)	Fish length^b^ (cm)	Sex^c^	GSI^d^ (%)
1^e^	39.8	29.96	120	F	0.0753
2	33.3	9.57	118	M	0.0287
3	34.6	14.55	118	M	0.0421
4	36.9	11.46	121	M	0.0311
5	36.3	16.81	121	M	0.0463
6	37.6	39.61	122	F	0.1053
7	48.7	21.8	131	M	0.0448
8	42.5	42.69	127	F	0.1004
9	36.6	56.76	123	F	0.1551
10	33.8	26.46	122	F	0.0783
11	36.2	12.27	122	M	0.0339
12	42.2	95.9	125	F	0.2273

^a^Gilled and gutted weight. ^b^Length was measured as total length (TL). ^c^M and F denotes male or female fish respectively. ^d^Gonado-somatic index (GSI), presented as percentage, was calculated as the ratio between gonad weight to gilled and gutted body weight (in grams). ^e^This fish was not used for RNA-Seq due to low quality RNA

All experimental procedures were performed in accordance with the University of the Sunshine Coast and Animal Ethics Committee guidelines (approval numbers AN/A/11/58), following the Australian Code for the Care and Use of Animals for Scientific Purposes [23].

### Sample preparation

The gonads were washed again in the laboratory in ice cold 10 mM PBS, weighed and sliced into 5 mm cross-sections. A whole cross-section slice from the middle part of each gonad was further cut into small pieces (5×5 mm) and stored in RNAlater (R0901; Sigma-Aldrich) at −20 °C until further processing. Testis sample from each male was further enzymatically dissociated, filtered and centrifuged through a discontinuous Percoll gradient (17-5445-01; GE Healthcare) to obtain ASG-enriched cell suspension, following the methods described in [11]. Enriched ASG cell suspensions were stored in 1 mL of RNAlater at −20 °C until further processing. Ovary sample from each female was dissociated using the same methods described above, however, oogonia enrichment was performed without Percoll gradient, but rather by consecutive filtering of the dissociated cell suspension through sterile 150 and 30 *μ*m filters (CellTrics 04-004-2326/9; Partec) respectively, as described in [24].

### Sample histology

Cross-section slices from each gonad were fixed in 4 % paraformaldehyde for 24 h, then dehydrated in graduated ethanol washes (30, 50, 70, 90, 95 and 100 % for 2 h at 4 °C) and xylene (twice for 40 min at room temperature). The cross-sections were finally embedded in paraffin (x3 times for 40 min at 65 °C) and mounted in blocks. Tissues in the blocks were sliced to 7 *μ*m sections using a microtome (UM-MS355; ProSciTech), and sections were then dried on superfrost slides (LBS4951; LabServ), stained with Harris hematoxylin and eosin (H&E) and permanently mounted using DePex (BDH Chemicals) [25].

Stained sections were visualized under a compound microscope (DM5500B; Leica Microsystems) and digital images were acquired using a digital camera (DFC550; Leica Microsystems) coupled with an image acquiring software (LAS v4.3; Leica Microsystems). Gonad maturation stage was assessed visually and fitted onto a categorical scale (ranging from 1–5 for testes and 1–9 for ovaries), based on the presence and morphology of germ cell populations in the gonads, as described in [26, 27].

### RNA extraction

RNA was extracted from five biological replicates (originating from different animals) of each group: enriched cells or crude extract from both testes and ovaries. Prior to RNA extraction, the samples were centrifuged at 4000 rpm for 3 min and the RNAlater supernatant was discarded by pipetting. Fifty milligram (50 mg) of crude gonad sample was placed in a 2 mL clean, RNase free micro-centrifuge tube, containing 1 mL RNAZol®; RT (RN 190; Molecular Research Center) and 5 ceramic (zirconium oxide) beads (2.8 mm, KT03961-1-002.2; Precellys). The tissue was homogenized using a Qiagen TissueLyser II (85300; Qiagen) at a frequency of 30 Hz, for 60 s, 2–6 times until no solid tissue was visible. The enriched cell suspensions were homogenized for 30 s using a motorized pellet pestle (Z359971; Sigma-Aldrich). Total RNA was then extracted according to the RNAZol® RT manufacturer instructions and stored at −80 °C. RNA quality and quantity were assessed visually using gel electrophoresis, then concentration was calculated from a spectrophotometer measurement (Nanodrop 2000; Thermo Scientific) and finally an RNA integrity number (RIN) was determined for each sample by a 2100 BioAnalyzer (G2947CA; Agilent).

### RNA-Sequencing

Best quality RNA extracts were selected from for both crude and enriched extracts of 5 female and 5 male gonads (*n* = 20) with RIN >6, preferably using gonad sample from the same individual fish for both crude and enriched preparation when RNA quality allowed. The RNA samples were then sent to the Australian Genome Research Facility (AGRF; Brisbane, QLD) for library preparation and RNA-Seq on an Illumina HiSeq2500 platform, producing 100 base pair (bp) paired end reads. All five replicates from each treatment were multiplexed on each lane, to a total of 4 lanes, achieving coverage of (8–12) × 10^6^ bp per sample.

#### Assembly

Short read sequences obtained from RNA-Seq were downloaded and processed in-house at the University of the Sunshine Coast. Preliminary quality check of the reads was performed using FastQC (v0.11.2) and further 3’ and 5’ ends quality trimming and Illumina adapters removal were performed by Trimmomatic (v0.32) [28]. The processed reads were mapped to a reference genome of the closely related PBT, *T. orientalis* [17], using BBMap spliced reads aligner (v33.65), with an adjusted seed length of *k* = 12 to account for cross-species mismatches. The resulting BAM files with the aligned reads were then used as an input for Trinity (r20140717) [29], to assemble the reads into a genome-guided *de novo* transcriptome of SBT gonad cells.

#### Gene and protein annotation

Open reading frames (ORFs) were predicted from the assembled transcripts using TransDecoder (r20140704); ORF was considered as complete by the presence of a starting methionine amino acid and an ending stop codon. Transcripts and predicted peptides were annotated by sequence alignment similarity search, BLAST (v2.2.30+) [30], to protein databases (NCBI nr, UniProt, Swiss-Prot and KOBAS, *e*-value <1×10^−3^) and by hidden Markov models protein domain identification using HMMER3.1 [31] against the HMMER/Pfam protein database (v27.0) [32]. Based on these annotations, Gene Ontology (GO) [33] and Kyoto Encyclopedia of Genes and Genomes (KEGG) [34] terms were assigned to each putative protein. Furthermore, prediction for presence of secretory signal peptide and trans-membrane topology were performed using SignalP (v4.1) [35] and TMHMM (v2.0) [36], respectively. The resulting annotation output files were further processed and cleaned to remove duplicates, select best matching annotation and identify errors or missing values.

#### Differential expression

The number of reads mapping to each transcript was estimated by RSEM [37]. Preliminary exploratory data analysis (EDA) of the estimated counts of each sample was performed by computing and plotting a between-samples distance matrix and principal component analysis (PCA) to identify sample-related bias. The estimated counts were fed to edgeR [38] to identify statistically significant differentially expressed (DE) transcripts between treatment groups. Both tools were initiated automatically using scripts from the Trinity pipeline. Transcripts were considered as DE when the absolute value of the log base-2 of the fold change was greater than 2 (|log_2_FC>2|, positive or negative for either over- or under-expression respectively) with an adjusted *p*-value <1×10^−3^ to reduce the false discovery rate(FDR). When multiple transcripts were annotated to the same DE gene, log_2_FC was calculated as the mean with corresponding standard errors (SE).

Gene set enrichment analysis (GSEA) was performed for the GO and KEGG annotated DE genes in each comparison using the goseqR package [39]. In brief, the total appearances of each GO and KEGG function was accumulated across DE genes, taking into account the length of each gene and determining enriched functions in each comparison group.

#### Data analysis

**Fig. 1:**
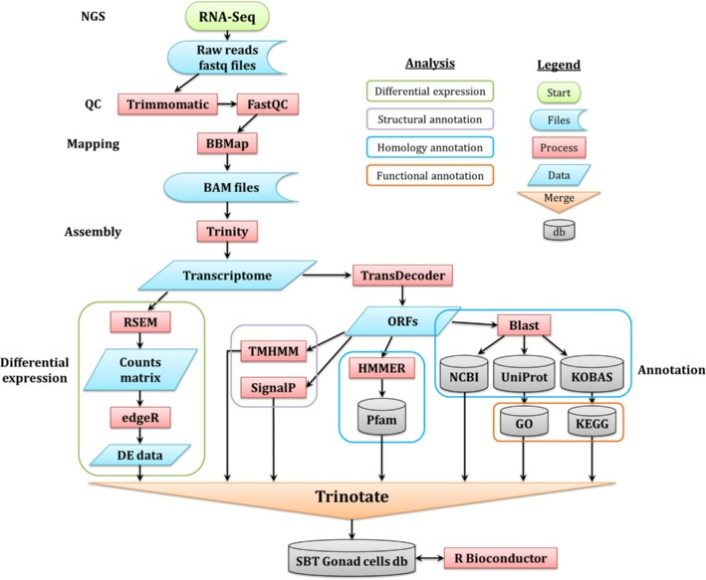
RNA-Sequencing data processing and analysis workflow

The annotation and expression data files were then combined and loaded onto a lightweight, standalone relational SQLite database (http://www.sqlite.org/), using the scripts provided in the Trinotate (v2.0.1, https://trinotate.github.io/) pipeline. This allowed fast and easy retrieval of sequences, annotation and expression data using any combination of conditional filtering and ordering. A complete bioinformatics data processing and analyses workflow is presented in Fig. 1.

Statistical analysis and further data summarizing was performed using the R (v3.1.2) statistical programming language [40]. Specifically, relevant data was retrieved from the SQLite database with RSQLite and sqldf packages and then further processed using various tools from the BioconductorR package repository (http://www.bioconductor.org/) [41].

#### Availability of supporting data

The data set containing the raw RNA-Seq reads generated in this study was deposited to the NCBI Sequence Read Archive database (study accession number: SRP059929), available at http://www.ncbi.nlm.nih.gov/bioproject/?term=PRJNA288188.

## Results and discussion

### Southern bluefin tuna gonad samples

**Fig. 2:**
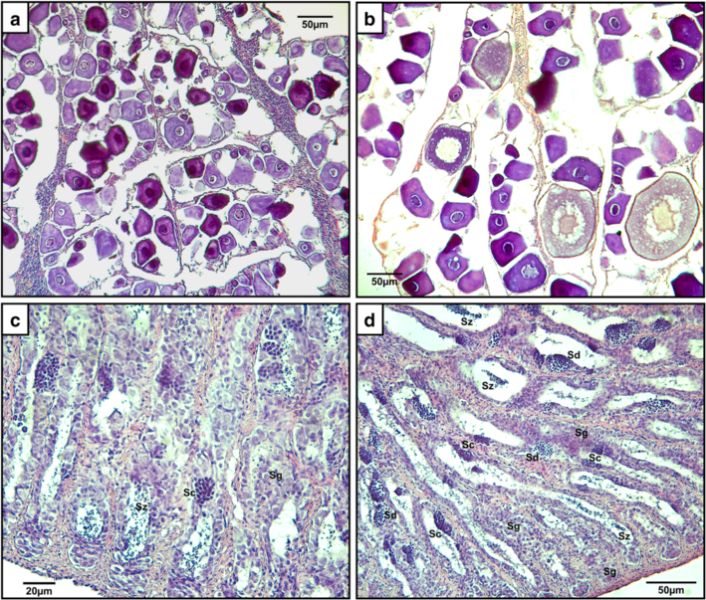
Southern bluefin tuna gonad histology. SBT ovary sections in maturation stages 1 and 3 are presented in images **a** and **b**, respectively. SBT testis in maturation stages 2 and 3 are presented in images **c** and **d**, respectively. Different spermatogonial germ cell populations are distinguished in images **c**–**d**: *Sg* – Spermatogonia types A and B (ASG/BSG), *Sc* – Spermatocytes, *Sd* – Spermatids, *Sz* – Spermatozoa. Sections were stained with H&E staining. 20x objective was used in all images. Detailed information regarding the image acquisition is provided in the ‘Methods’ section

Twelve SBT were sampled on May 27, 2014 from Cleanseas Tuna Ltd. sea-pens located in Port Lincoln bay, South Australia. Females mean body weight was 38.75 kg ± 340 kg with a mean GSI of 0.124 % ± 0.058 %; males mean body weight and GSI were 37.67 kg ±5.56 kg and 0.0378 % ± 0.0075 % respectively (*n* = 6 for each sex, Table 1). Histology of the sampled fish gonads revealed that the female ovaries show an asynchronous development pattern and were categorized at stages 1–3 (on a scale of 1–9 [27]) and comprised mainly of unyolked oocytes up to 50 μm in diameter, with a few early yolked oocytes up to 130 μm, in diameter in the more developed ovaries (Table 1 and Fig. 2a–b). The males were at stages 2–3 (on a scale of 1–5 [27]), displaying all germ cell populations, from ASG to spermatozoa; however, the majority of the germ cells were at early stages of spermatogonia with increasing number of spermatocytes in stage 3 testes (Table 1 and Fig. 2c–d). These body weights, GSI and gonad maturation stages are consistent with the previously reported range of values for early maturing male and sexually immature female SBT. For example, females have been reported as being sexually immature at GSI <0.22 % and gonad development stages below 4, while males start maturing at GSI <0.06 % and gonad development stage 3 [27].

**Fig. 3:**
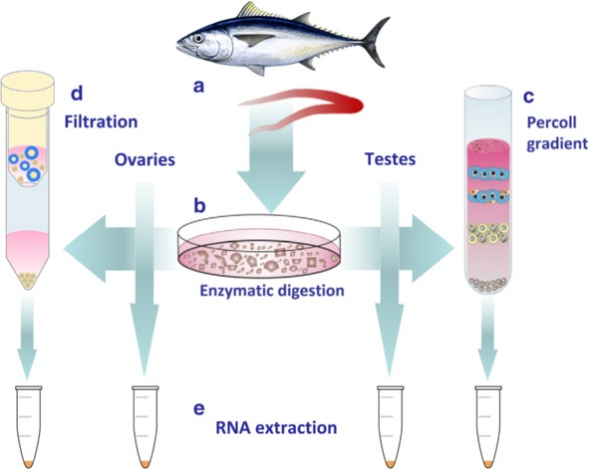
Preparation of Southern bluefin tuna gonad samples for RNA extraction. **a** – Gonad dissection (ovaries from females and testes from males); **b** – Enzymatic digestion of the crude gonads to break down the tissue to individual cells; **c** – Percoll gradient enrichment of ASG from the testes cells; **d** – Filtration through a 30 *μ*m pore size filter to enrich oogonia cells from the ovary cells; **e** – RNA extraction from each of the 4 groups (testes/ovaries, both crude/enriched)

Gonad sampling, preparation and RNA extraction workflow is presented in Fig. 3. Testis samples produced higher quality RNA following extraction, with average RIN of 7.8 for the crude extracts and 9.12 for the Percoll-enriched cell extracts, whereas ovary cells crude extracts RIN averaged at 7.1 (Table 2). A RIN for the filtered ovary cells extract could not be determined due to a low molecular weight peak present in the gel electrophoresis. The same artifact affected the crude ovary cells extract as well, resulting in the observed lower RIN numbers compared with testis cells extracts. All attempts to extract higher quality RNA without this artifact have failed.

**Table 2:** Gonad cell samples, treatment and RNA extraction quality and quantity details

Sample^a^	Tissue	Preparation treatment	RIN^b^	RNA conc. (ng/*μ*L)
SBT_T2	Testis	Crude	8.10	100.00
SBT_T3	Testis	Crude	8.00	100.00
SBT_T4	Testis	Crude	8.50	100.00
SBT_T7	Testis	Crude	7.20	100.00
SBT_T11	Testis	Crude	7.30	100.00
SBT_TP2	Testis	Enriched	9.10	40.00
SBT_TP3	Testis	Enriched	9.60	100.00
SBT_TP4	Testis	Enriched	8.90	100.00
SBT_TP5	Testis	Enriched	9.30	100.00
SBT_TP7	Testis	Enriched	8.70	100.00
SBT_O6	Ovary	Crude	6.50	100.00
SBT_O8	Ovary	Crude	7.10	100.00
SBT_O9	Ovary	Crude	6.50	100.00
SBT_O10	Ovary	Crude	7.80	100.00
SBT_O12	Ovary	Crude	7.60	100.00
SBT_OC6	Ovary	Enriched	NA	100.00
SBT_OC8	Ovary	Enriched	NA	100.00
SBT_OC9	Ovary	Enriched	NA	100.00
SBT_OC10	Ovary	Enriched	NA	100.00
SBT_OC12	Ovary	Enriched	8	85.00

^a^Sample name explanation: letters after the underscore stand for the tissue the cells derived from: ‘T’ – Testis; ‘TP’ – Testis cells enriched with Percoll; ‘O’ – Ovary; ‘OC’ – Ovary filtered Cells. The number at the end of each sample id correlates to fish id in Table 1. ^b^RNA integrity number (RIN), as determined by 2100 BioAnalyzer

Apparently, fish ovarian RNA contain a high peak of 5S ribosomal RNA, which changes the RNA profile on the gel and interferes with correct estimation of the 18S and 28S rRNA peaks, resulting in poor RIN numbers [42, 43]. Despite these abnormalities in the quality of the ovary cell RNA extracts, the RNA samples did pass the required quality checks at the RNA-Seq provider and were further processed and sequenced.

### Southern bluefin tuna gonadal cells transcriptome assembly

**Fig. 4:**
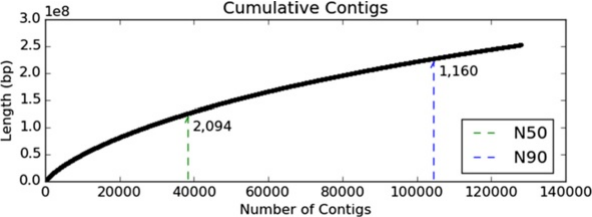
Cumulative assembly contigs showing N50 and N90 statistics. Length in Y-axis is presented in 100 million base pairs (1×10^8^ bp)

A combined total of 1.55×10^9^ short reads, 100 bp each, were sequenced by the Illumina Hiseq2500 RNA-Seq from all the samples and all treatments; of those reads, 95.4 % (1.48×10^9^ reads) passed adapter removal, length and quality control (QC). The raw reads that passed QC were mapped to the genome of the closely related PBT (*T. orientalis*), giving an average mapping rate of 95.51 % and resulting in 1.34×10^9^ mapped reads, an average of 6.72×10^7^ reads/sample. The mapped reads were then assembled using a combined approach of genome-guided and *de novo* assembly, as detailed in Methods subsection ‘Assembly’, resulting in 128,065 contigs, with a median of N50 = 2094 bp and a longest contig of 20,234 bp long (Table 3 and Fig. 4).

**Table 3:** Southern bluefin tuna gonadal cells transcriptome assembly details

Feature	Value
Assembled contigs (n)	128,065
Longest contig (bp)	20,234
Mean contig (bp)	1973
Contig N50 (bp)	2094
Contig N90 (bp)	1160
Total contig length (bp)	2.53×10^8^
Assembly GC content (%)	42.22
Reads passed QC^a^ (n)	1.48×10^9^
Reads failed QC (n)	7.13×10^7^
Mapped reads (n)	1.34×10^9^
Average mapping rate (%)	95.51

^a^Quality control (QC) of the raw sequenced reads was determined by FastQC

When attempting to assemble the RNA-Seq transcriptome of a non-model species, whose genome is not available, two options for assembly can be considered: 1) A *de novo* approach, where transcripts are built solely from overlapping segments in the reads [29, 44–46]; or 2) A genome-based approach, where reads are aligned to a reference genome of a closely related species [47, 48]. A genome-based mapping can produce longer contigs and wider coverage thanks to the ability to close gaps of low read coverage. However, it uses a more lenient mapping strategy (smaller k-mer size compared with a *de novo* approach) to account for mismatches between the species, thus increasing the chance of introducing artifacts and sequencing errors [47]. This trade-off must be considered when deciding which strategy to utilize, depending on the aim of the study. In this study, a combined approach was chosen, using the genome of the closely related PBT as a reference and mapping the reads to it followed by a *de novo* assembly while taking into account sequence dissimilarities and novel transcripts [29, 47, 49–51]. This strategy was selected to discover as many genes as possible that are involved in the reproduction and gonad development in the SBT, while using tools like homology-based annotation and coding region (CD) prediction to assist in quality filtering of the transcriptome. When higher fidelity is required, for example when the focus of the research is on discovery of isoforms and variant detection, a *de novo* approach with higher stringency setting might be more suitable [47].

### Transcript annotation

**Fig. 5:**
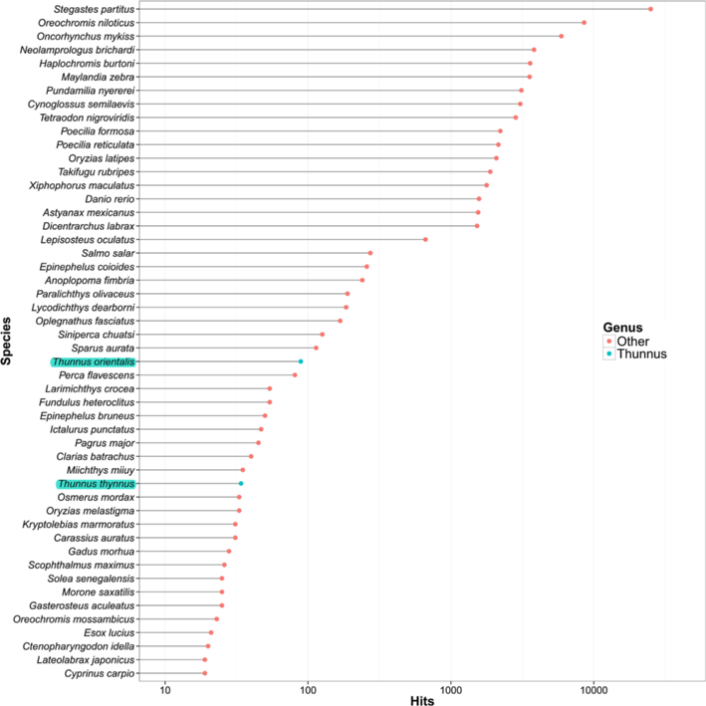
The 50 most abundant Blast annotated taxonomy species. ORFs were annotated by sequence similarity against the NCBI non-redundant (nr) database, with an *e*-value <1×10^−3^. Top 50 most annotated species are presented. *Thunnus* species are highlighted. X-axis is in logarithmic scale

Multiple ORFs were predicted from each transcript, to a total of 136,465 ORFs. The ORF list was further processed to remove redundancies (ORFs sharing exactly the same peptide sequence) and then annotated to known proteins. For each ORF, the best hit annotation was selected, discarding ORFs that had no hit within any of the protein databases used, to provide a final list of 84,204 annotated ORFs from 300 different species (Table 4). Only 145 out of the 84,204 ORFs (less than 0.2 %) were annotated back to species of the *Thunnus* genus (highlighted in Fig. 5). The low number of ORFs annotated to *Thunnus* genus emphasizes the lack of publicly available annotated tuna gene data. Currently, 6754 nucleotide and 2120 protein sequences from the entire *Thunnus* genus (taxonomy id: 8234) are available on NCBI databases. These include mainly mitochondrial DNA used in phylogenetic and energy metabolism studies (approximately 50 % of the sequences), as well as genes involved in the optic, locomotion and reproductive systems, the main functional systems studied in the tunas [17, 52–55]. These sequences contain a high level of redundancy, with multiple isoforms and partial sequences for each gene.

**Table 4:** Open reading frame prediction and annotation

Feature	Occurrences	Annotated^b^
Total predicted ORFs^a^	13,6465	
Non redundant (NR) ORFs	119,361	84,204
Complete NR ORFs	36,493	18,210
5’ Partial NR ORFs	31,905	22,175
3’ Partial NR ORFs	19,209	15,785
Internal partial NR ORFs	31,754	28,034
ORFs taxonomic species hits		300

^a^ORFs predicted by TransDecoder and considered as complete based on the presence of a starting methionine and an ending stop codon. ^b^ORFs were annotated by either the NCBI non-redundant (nr), Uniprot, KEGG or Pfam protein databases, with an *e*-value <1×10^−3^

### Differential expression exploratory data analysis

Differentially expressed (DE) gene analysis was performed on the estimated counts of the reads mapped to the CD of predicted ORFs of each transcript; the count estimation was performed by RSEM software.

**Fig. 6:**
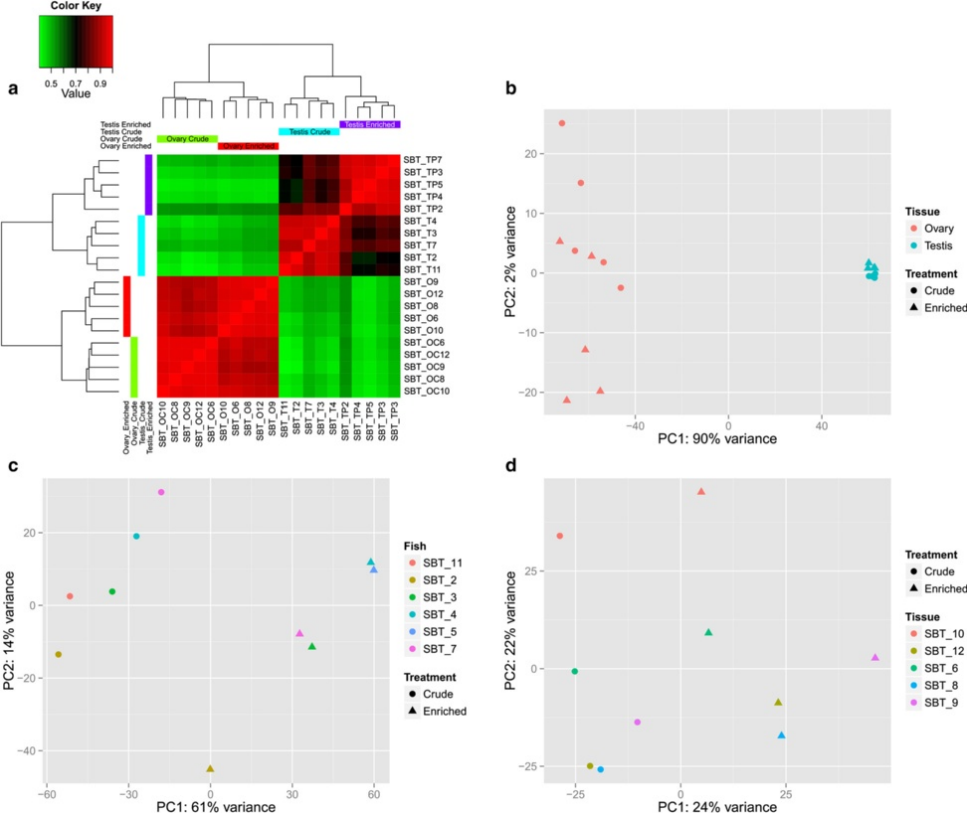
Exploratory data analysis of differentially expressed genes in SBT gonad cells across all comparison groups. **a** – Cluster analysis of SBT gonad samples. Red color in matrix represents no difference between samples and green represents maximum distance; Colored bars on top and left of figure represent one treatment group each: green for enriched ovary cells (labeled SBT_OC), red for crude ovary cells (labeled SBT_O), light blue for testis cells (labeled SBT_T) and purple for Percoll-enriched testis cells (labeled SBT_TP). **b** – Principal component analysis (PCA) of regularized log-transformed counts in all samples to identify the effects of germ cell enrichment treatment and gonad type (testis or ovary). **c** – PCA of regularized log-transformed counts in testis samples to identify the effects of the Percoll enrichment treatment and individual fish across samples. **d** – PCA of regularized log transformed counts in the ovary samples to identify the effect of the filtration enrichment treatment and individual fish across samples

Prior to the DE gene analysis, an EDA was performed to detect major factors or biases affecting the variability between the different analysis groups and samples. A distance matrix was computed between each sample/treatment combination and similar combinations were clustered together, as presented by the heatmap and dendrogram in Fig. 6a. The samples from each replicate cluster together by the tissue type and treatment: testis cells, Percoll-enriched testis cells, ovary cells and enriched ovary cells. The greatest variation was found between testis cells and ovary cells, both crude and enriched (green squares in top left and bottom right of Fig. 6a). Medium variation was found between the Percoll-enriched testis cells and the crude testis cells extracts (black squares in top right of Fig. 6a). The least variation was found between enriched ovary cells and crude ovary cells extracts (dark red squares in bottom left of Fig. 6a).

Principal component analysis (PCA) was performed on the regularized log-transformed counts, using tissue and treatment (crude/enriched) as factors (Fig. 6b). The tissue factor was found to contribute to 90 % of the variance, whereas the treatment factor for only 2 %. The testis cell samples, both crude and enriched extracts grouped tightly together, with a small but distinct difference between the crude and the Percoll-enriched cells. The ovary cell samples, however, were widely scattered with less obvious separation between the 2 treatments.

A second PCA was performed, this time for each tissue separately and including the individual fish which the samples were taken from as a factor. The testis cells showed a similar pattern as observed in the first analysis, with clear separation between crude and enriched cell samples, meaning that the enrichment treatment contributed to 61 % of the variance, with no significant effect of the individual fish (Fig. 6c). The ovary cells exhibited some separation by treatment (x-axis in Fig. 6d); however, the individual fish that each sample originated from had a significant contribution to the variance, at the same level as the treatment effect (y-axis in Fig. 6d).

The results of the EDA suggest that the sampled male fish were more uniform in their gonadal cell expression profile (Fig. 6c) and shown a significant effect caused by the Percoll enrichment treatment, whereas the sampled females exhibited an individual-dependent expression profile (Fig. 6d), with little effect caused by the filtration enrichment treatment. This suggests that the Percoll enrichment protocol used for the testis cells was more effective and produced a significant difference in the composition of the resultant cell suspension, whereas the female ovary cell filtration had little (if any) germ cell enrichment effect. An alternative explanation might be that the sampled female fish had inherently greater gonadal development variability between individuals than that of the sampled males. The GSI distribution of the sampled females supports the latter, with a standard error which accounts for 46 % of the mean (0.124 % ± 0.058 %), while in the males, the GSI standard error was less than 20 % of the mean (0.0378 % ± 0.0075 %), as detailed in Table 1. Both male and female GSI values were below the reported minimum for sexually mature SBT [27]. High variability within groups in gene expression data of fish gonads was reported in similar RNA-Seq transcriptome analysis of the sharpsnout seabream (*Diploduspuntazzo*) [43].

The filtration method used to enrich undifferentiated oogonia cells was based on physical dimensions of the pre-vitellogenic oogonia, which are the smallest germ cells in the fish ovary [24, 56]. This method was used after a failed attempt to obtain sufficient numbers of oogonia when using the same Percoll enrichment method that was used to enrich spermatogonia from testis. However, the ovary cell count data EDA demonstrated high variability within treatment groups and non-significant effect of the filtration enrichment treatment, which contributed to just over 20 % of the count data variance (according to the PCA of the ovary cell samples, Fig. 6d). Based on these results, it was concluded that any DE transcripts found in this comparison could not be accounted for the enrichment procedure and therefore this comparison was discarded from the subsequent DE transcript analysis.

These results, along with the lower RNA quality of the enriched ovary cell extractions (Table 2), suggest that an improved method for oogonia separation is needed, both for GCT and for comparative gene expression purposes.

### DE genes in crude ovary *vs.* testis cells

**Fig. 7:**
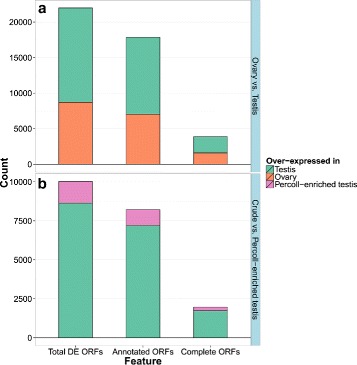
A summary of differentially expressed ORFs in Southern bluefin tuna gonadal cells transcriptome. The number of DE ORF features in each comparison (**a** - Crude ovary vs. testis cells and **b** - Crude testis *vs*. Percoll-enriched cells). The features are hierarchical, i.e., the complete and annotated ORFs are included in the total DE ORFs and might overlap. The number of over-expressed genes in each gonadal cell extraction is color-coded within each feature

DE transcript analysis was performed on a pairwise comparison (contrasts) of crude ovary cells *vs.* crude testis cells. The resulting DE transcripts of each analysis were filtered to include only annotated ORFs that passed the thresholds of |log_2_FC >2| (positive or negative for either over- or under-expression, respectively) and *p*-adjusted-value <1×10^3^. The average rate of annotated ORFs out of the total DE ORFs in this comparison was 81.2 % and these will be referred to from now on as “genes”. The number of DE genes in each tissue/treatment combination within each analysis and the ratio between annotated and non-annotated ones are presented in Table 5 and Fig. 7a.

**Table 5:** Differentially expressed (DE) genes summary

Analysis subset	Total DE genes^a^	Annotated ORFs^b^	Complete annotated ORFs
Ovary *vs.* testis cells	21,984	17,849	3868
Overexpressed in ovary cells	8706	7026	1587
Overexpressed in testis cells	13,278	10,823	2281
Crude *vs.* enriched testis cells	10,026	8220	1970
Overexpressed in crude testis cells	8637	7205	1732
Overexpressed in enriched testis cells	1389	1015	238

^a^Genes represented as open reading frame coding regions (ORF-CDs) and were considered as significantly DE with a log_2_FC>2| and *p*-adjusted-value <1×10^−3^. ^b^ORFs were annotated by either the NCBI non-redundant (nr), Uniprot, KEGG or Pfam protein databases, with an *e*-value <1×10^−3^

Altogether, 17,849 annotated ORFs were found to be DE when comparing ovary to testis cells; 10,823 of them over-expressed in the testis and just over 7000 were over-expressed in the ovary cells. The total number of DE genes and the bias towards over-expression in testis match reported results of DE gene analysis in gonads of Nile tilapia (*Oreochromis niloticus*) [57] zebrafish (*Danio rerio*) [58, 59] and sharpsnout seabream (*D. puntazzo*) [43], where similar differences were found. According to these studies, this bias towards male transcripts had been suggested to occur as a result of evolutionary pressure, driven by male competitiveness.

**Fig. 8:**
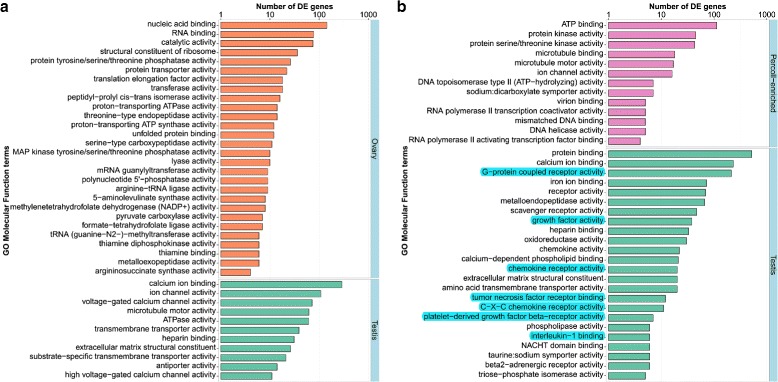
Gene Ontology molecular function enrichment in each comparison (**a** - Crude ovary *vs*. testis cells and **b** - Crude testis *vs*. Percoll-enriched cells). The number of genes from each molecular function that were over-expressed in each tissue (with a log_2_FC>2). X-axis is in logarithmic scale

Each gene was further annotated by assigning GO terms associated with its UniProt and Swiss-Prot annotation. Similar GO molecular function terms were then clustered together and a DE GSEA was performed to determine enriched terms in each analysis set (Fig. 8, full list available in Additional file 1). Enriched GO molecular functions with a *q*-value <0.01 in the ovary *vs.* testis cells DE analysis are presented in Fig. 8, separated by the tissue in which each term is over-expressed. Review of these DE enriched GO terms reveals that the majority are general cell maintenance molecular functions related to metabolism and protein translation and synthesis, such as nucleic acid and RNA binding and ATPase activity. In contrast to the ovary, testis cells contain an abundance of genes with motility-related molecular functions.

**Fig. 9:**
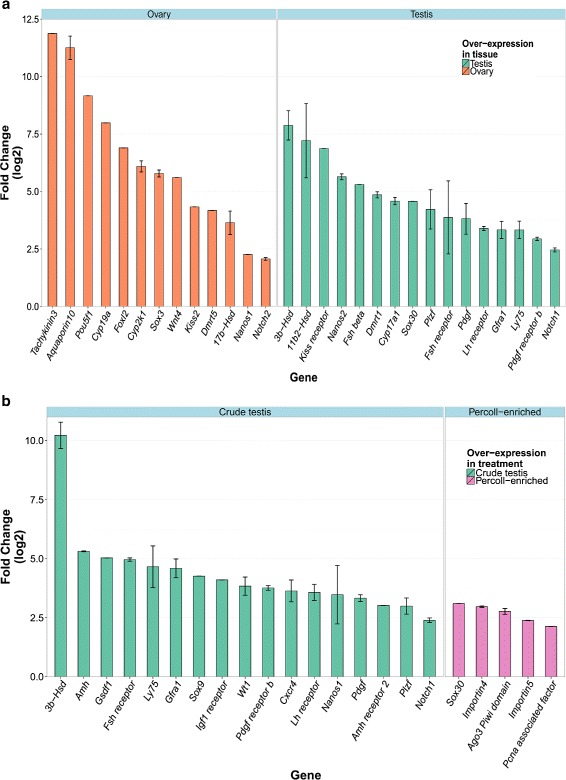
Differentially expressed gonad development and germ cell-related genes in Southern bluefin tuna gonadal cells. **a**–Ovary *vs*. testis cells comparison. **b**–Crude testis *vs*. Percoll-enriched cells comparison. When multiple transcripts were annotated to the same DE gene, log_2_FC was calculated as the mean with corresponding standard error (SE) bars

An *in silico* enrichment of specific germ cell and gonadal development-related genes and markers was used to refine the DE analysis results. An in-depth analysis of a subset of germ cell and gonadal development-related genes revealed additional expression patterns (selected genes are presented in Fig. 9 and a full list in Additional file 2) as detailed below.

#### Steroid synthesis related genes

Tissue-specific (and consequently sex-specific) DE steroid synthesis genes are notable: 3- *β*-hydroxysteroid dehydrogenase (*3b-hsd*), expressed in Leydig cells and responsible for biosynthesis of steroids, mainly progesterone and testosterone [60], was highly over-expressed in testis cells. On the other hand, 17- *β*-hydroxysteroid dehydrogenase 8 (*17b-hsd8*) and *17b-hsd12*, that regulate the concentration of biologically active estrogens and androgens and are involved in synthesis of estradiol (E2) [61], were both over-expressed in ovary cells.

Corticosteroids and their receptors play an important role in regulating reproductive functions by acting as transcription factors in the gonad somatic cells [62]. A member of the corticosteroids synthesis pathway, which was found to be over-expressed in high levels in testis cells, is 11- *β*-hydroxysteroid dehydrogenase 2 (*11b2-hsd*), an enzyme which converts gonad maturation inhibiting cortisol or corticosterone into inactive cortisone or 11-dihydrocorticosterone respectively [62, 63].

Other genes involved in the synthesis of steroids from cholesterol are the P450 cytochrome enzymes. The 17- *α*-hydroxylase, 17, 20 lyase (*cyp17a1*) was over-expressed in the testis cells, whereas aromatase (*cyp19a*) was highly over-expressed in ovarian cells. *cyp17a1* and *cyp19a* were shown to express both in germ cells and Leydig cells in zebrafish (*D. rerio*) testis, with high expression levels of *cyp17a1* in Leydig cells. However, exposure to exogenous steroids, such as E2, can dramatically lower the expression levels of *cyp17a1* in Leydig cells and lead to a disruption of spermatogenesis [64].

A comparison between XX female to XY male Nile tilapia (*O. niloticus*) gonads found similar expression patterns to the ones detailed above for *3b-hsd*, *17b-hsd8*, *17b-hsd12a1*, *11b2-hsd*, *cyp17a1* and *cyp19a* [65, 66]. The *cyp2k1*, which was highly over-expressed in the ovary cells, is known for its lauric acid (*ω*-1) and (*ω*-2), E2 and progesterone hydroxylation activities [67, 68]. It had also been suggested as a biomarker to determine prior exposure to produced formation water, that is, water that are discharged from oil-well platforms and produced as a by-product along with the oil [69]. Interestingly, these oil-well platforms are located at the North-West Shelf of Australia, on the migration route of SBT, between the only reported spawning grounds in South-East of Java, Indonesia [70] and their site of capture for ranching in the Great Australian Bight in South Australia.

The above mentioned steroid synthesis genes demonstrate an expected expression pattern, with androgen synthesis enzymes over-expressed in the testis cells, while estrogen synthesis enzymes over-expressed in the ovary cells. The function of *cyp2k1* has not been well defined in the context of sex steroids synthesis, but rather in the metabolism of carcinogenic substances, and therefore its high level of over-expression in the ovary cells requires further investigation.

#### Sex-specific genes

A second set of genes that were DE between ovary and testis cells are genes associated with sex determination and sex-specific gonad development. One such gene that was highly expressed in the testis cells is the DNA binding motif domain Double-sex and mab-3 related transcription factor 1 (*dmrt1*). The *dmrt1* is known to be involved in a male-specific vertebrate sex-determining pathway and is the key sex-determining factor in medaka (*Oryzias latipes*) [71–73], with similar function suggested in tilapia (*O. niloticus*) [66] and rainbow trout (*Oncorhynchus mykiss*) [74]. In contrast, *dmrt5*, a less studied member of the *dmrt* family, was found to be over-expressed in the ovary cells. In zebrafish (*D. rerio*), *dmrt5* was shown to be involved in the endocrine control of the reproductive system by controlling corticotrope and gonadotrope differentiation in the pituitary [75]. In zebrafish gonads, *dmrt5* expression was limited to developing germ cells, especially spermatogonia, spermatocytes, spermatids and sperm cells in the testis; and developing oocytes, including early perinucleolus stage oocyte, late yolk vesicle stage oocyte, and oil drop stage oocyte in the ovary [76]. Its expression pattern in the SBT therefore could be indicative of the developmental stage of the gonad.

The SRY-related HMG-box 3 (*sox3*) transcription factor, is often mentioned along with *dmrt1* as a male-specific gene, involved in early stages of testis development and growth, mainly in mammals [77, 78], but also in sex-changing protandrous (*Acanthopagrus schlegeli*) and protogynous (*Epinephelus coioides*) fish [79, 80]. In SBT however, *sox3* was found highly over-expressed (log_2_FC=5.8) in the ovary cells, indicating it might have additional roles in ovarian differentiation not previously recognized. Another Sox transcription factor gene, *sox30*, had an opposing expression pattern and was moderately over-expressed (log_2_FC=4.6) in testis cells. Just recently identified in non-mammalian vertebrates, *sox30* was found to be expressed exclusively in germ cells within Nile tilapia (*O. niloticus*) testis, indicating its possible involvement in spermatogonial differentiation and spermatogenesis, like in human and mouse [81, 82].

The wingless-type MMTV integration site family member 4 (*wnt4*) gene, was moderately over-expressed in the ovary cells (log_2_FC=5.6). *Wnt4* is one of the few genes that were identified as ovarian-determining genes in mammals; it regulates the development of the female reproductive tract by suppressing male features development in XX gonads [83–85]. In fish, *wnt4* has been reported to be important to oocyte maturation and is expressed in the developing ovary of both protandrous (*A. schlegeli*) and protogynous (*E. coioides*) fish [86, 87]. Similar to *wnt4*, the forkhead-box L2 (*foxl2*) transcription factor, which was highly over-expressed (log_2_FC=6.9) in the SBT ovary cells, has been associated with female gonad differentiation through its function as a transcriptional regulator of aromatase (*cyp19a*), a function that is conserved across a variety of vertebrates, including mammals and fish [84, 88]. In fish, *foxl2* was shown to be expressed exclusively in somatic cells of developing (pre-vitellogenic) ovaries of gonochoristic fish such as tilapia [66, 89], medaka (*O. latipes* and *Oryzias luzonensis*) [90, 91], Japanese flounder (*Paralichthys olivaceus*) [92] and Rare minnow (*Gobiocypris rarus*) [93] to name a few. In sex-reversing hermaphrodite fish, however, *foxl2* expression pattern was not as strictly defined: it was still highly expressed in early ovarian developmental stages gonads of protogynous species (*E. coioides*, *Halichoeres trimaculatus* and *Monopterus albus*) [79, 87, 94, 95] but its expression levels declined in transition to testis, and *vice versa* in protandrous species (*A. schlegeli* and *D. puntazzo*) [43, 86].

#### Endocrine regulation-related genes

Another set of genes that were found to be over-expressed in testis cells compared to ovary cells included various gonadotropin pathway receptors: luteinizing hormone (*lh*) receptor, follicle stimulating hormone (*fsh*) receptor and gonadotropin releasing hormone (*gnrh*) receptor. These genes were moderately over-expressed in the testis (log_2_FC up to 5), along with high over-expression (log_2_FC=6.87) of the kisspeptin1 receptor (*kiss1r*). This synchronous expression pattern was previously reported during testis germ cell maturation in the fathead minnow (*Pimephales promelas*) [96] and the Scombrid chub mackerel (*Scomber japonicus*) [97–100]. The platelet-derived growth factor (*pdgf*) and its receptor (*pdgf receptor b*) were both moderately over-expressed (log_2_FC between 3 and 4) in the testis cells. The *Pdgf* signaling system, through its several isoforms and receptors, has been shown to play a critical role in regulating the development and functional control of various tissues in humans and mice [101, 102]. In testicular development, *pdgf* was shown to be expressed by Sertoli cells to promote proliferation and/or differentiation of Leydig cells, and is required for intra-gonad cell migration, both in fetal and adult testis [83, 103].

In rainbow trout (*O. mykiss*) developing embryos, *pdgf receptor* expression was found to be in tight association with *dmrt1* and therefore was suggested as a male sex-determining gene [74].

Another genes of interest are *tachykinin3*, which encodes a neuropeptide that plays an important role in neuroendocrine regulation of reproduction and its associate *kisspeptin*, known as the puberty onset gene in mammals, where it controls pulsatile gonadotropin-releasing hormone release [3, 104]. Both *tachykinin3* and *kisspeptin* were over-expressed in the SBT ovary cells. While *tachykinin3* displayed some of the highest over-expression (log_2_FC=11.88) among the gonad development-related genes in the ovary, *kisspeptin* was moderately over-expressed (log_2_FC=4.33) in an interesting contrast to the high over-expression of its receptor in the testis.

A member of the Notch protein family, *notch2*, was slightly over-expressed in the ovary cells. Involvement of Notch2 protein in ovarian development has not been thoroughly studied in fish so far; in mouse however, it was recently suggested that *Notch2* cooperates with gonadotropins to regulate ovarian follicle formation and growth [105, 106]. This finding in SBT suggests it might have a similar role in fish.

#### Germ cell-specific genes

A final set of genes that were DE in this comparison are germ cell-specific genes. The lymphocyte antigen 75 (*ly75*), glial cell line-derived neurotrophic factor (GDNF) family receptor alpha 1 (*gfra1*), *nanos2*, *notch1* and promyelocytic leukaemia zinc finger (*plzf*), which were previously reported as ASG-specific markers in GCT studies [107], were over-expressed in the testis at moderate levels (log_2_FC between 2.5 and 5).

The POU domain, class 5, transcription factor 1 (*pou5f1*), also known as octamer-binding transcription factor 4 (*oct4*) and *nanos1* are key genes in stem cell maintenance and regulation [108–110]. Both were over-expressed in the ovary, with very high expression levels of *pou5f1* (log_2_FC=9.15), indicating strong stem cell activity in the female ovaries.

The *aquaporin10* gene, encoding for a water (and other solubles) channel, was found at very high levels at the ovary cells (log_2_FC=11), an approximate x2000 fold change compared with its expression in testis cells. Similar expression pattern (though with lower fold change) was reported in a comparison of gene expression levels between mature ovaries and testis of BFT (*T. thynnus thynnus*) [7]. Aquaporin10 has been reported to facilitate oocyte hydration in the maturation of buoyant (pelagic) eggs of the gilthead sea bream (*Sparus aurata*); though oocyte hydration occurs at late stages of oocyte maturation, *aquaporin10* was highly expressed in early vitellogenic ovaries [111, 112].

#### Crude ovary *vs.* testis cells DE summary

The gonad development gene expression patterns detailed above suggest that the ovaries of the sampled female fish were at an early stage of gonadal development, mainly expressing high levels of transcripts for stem cells maintenance (*nanos1* and *pou5f1*/*oct4*) and early stages of oocyte development-related genes (*aquaporin10*, *foxl2* and *dmrt5*). In contrast, genes identified as over-expressed in testis cells, encode for proteins whose function indicates a more advanced gonad developmental stage, with increased expression of receptors for reproductive hormones (*fsh*, *lh*, *gnrh* and *kisspeptin* receptors) as well as enzymes involved in sex steroids and hormone synthesis and signaling.

These findings are supported by the previously determined minimal weights for sexual maturation in SBT, in which males display sexual maturation earlier than females, at a minimal weight of 50 kg and GSI >0.065*%*, while females exhibit sexual maturation signs only from a weight of 100 kg and GSI >0.71 *%* [27]. The sexual maturation process in SBT males progresses at a faster rate than in females, with all stages of germ cells (ASG, type-B spermatogonia - BSG, spermatocytes, spermatids and spermatozoa) observed already in immature males in the second stage of spermatogenesis (observed in males weighing 30–71 kg) [27]. The fish that were sampled in this study, in comparison, weighed 38 kg on average, with the heaviest male weighing just under 50 kg, with a GSI =0.046 *%*, determined to be at stage 3 (out of 5) of spermatogenesis (early maturing testis) [27]. The heaviest female weighed 42.2 kg, with a GSI =0.22 *%* and determined to be at stage 3 (out of 9) of gonad development (immature ovary, details in Table 1 and Fig. 2) [27]. These results confirm that the males were approaching minimal size for sexual maturation, whereas the females were still in early stages of gonadal development and in preparation for puberty.

### DE genes in crude *vs.* Percoll-enriched testis cells

DE gene analysis was performed on a pairwise comparison (contrasts) of crude testis cells *vs.* Percoll-enriched testis cells. The resulting DE transcripts of each analysis were filtered as described for the crude testis *vs.* crude ovary (subsection ‘DE genes in crude ovary *vs.* testis cells’). The average rate of annotated ORF out of the total DE genes in this comparison was 82 %. The number of DE genes in each tissue/treatment combination within each analysis and the ratio between annotated and non-annotated ones are presented in Table 5 and Fig. 7b. The genes were further annotated by GO terms and then analyzed as previously described.

GSEA of GO molecular functions associated with DE genes in crude testis compared with Percoll-enriched testis cells was performed. The genes that were found over-expressed in Percoll-enriched cells are mainly involved in transcript translation (RNA polymerase and DNA binding) and cell motility (microtubule, ion channels and ATPase activities) (Fig. 7b, full list available in Additional file 3). Similar enrichment of these functions was observed in microarray gene expression of rainbow trout (*O. mykiss*) testis cells; specifically, enrichment of genes related to RNA metabolism and DNA replication functions was associated with advanced-stage, proliferating spermatogonia, whereas cell motility functions were enriched in post-meiotic testicular germ cells [113]. Within the molecular functions enriched in the crude testis cells, a few functions related to germ cell activity stand out: G-protein coupled receptors, growth factor, chemokine receptor, tumor necrosis factor receptor, platelet-derived growth factor receptor and interleukin 1 binding activities (highlighted in Fig. 7b). These molecular functions were reported to be associated with somatic cells regulation of spermatogonia maintenance and differentiation [107, 113, 114].

An *in silico* enrichment of gonad development and germ cell-related genes was performed to reveal specific expression patterns out of the entire DE genes in the crude *vs.* Percoll-enriched testis cells (selected genes are presented in Fig. 8b and a full list in Additional file 4).

#### Germ cell-specific genes

A set of germ cell-specific genes was found to be over-expressed at the crude testis cells when compared to the Percoll-enriched cells (Fig. 8b). These include the genes *ly75*, *plzf*, *notch1*, *nanos1*, *gfra1*, chemokine receptor 4 (*cxcr4*), platelet-derived growth factor receptor (*pdgf receptor*) and Wilm’s tumor suppressor 1 (*wt1*), all previously reported to be expressed in ASG and involved in germ cells maintenance [107, 115]. In human, *WT1* encodes a zinc finger transcription factor and RNA-binding protein that can activate or repress numerous target genes, including *PDGF*, thus playing a key role in testis development and maintenance [115, 116]. The G-protein-coupled chemokine receptor Cxcr4 is expressed in germ cells and directs their migration to the genital ridge in the developing embryo through detection and binding of its ligand, Sdf1 (Cxcl12) [117, 118]. The Cxcr4–Sdf1 signaling system has been found to be highly conserved across fish and other vertebrate species, enabling the migration of transplanted germ cells to the genital ridge of host larvae in cross-species GCT experiments [119].

In contrast, importins 4 and 5 (*ipo4/5*) were slightly over-expressed (log_2_FC of 3 and 2.4, respectively) in the Percoll-enriched testis cells. Importin proteins are nucleo-cytoplasmic transport proteins that play a central role in cargo-specific protein transport through the nuclear pores and into the nucleus, thus tightly regulating access of transcription factors to the genomic DNA. In mammalian testis, *Ipo4* and *Ipo5*, both importin- *β* family members, were up-regulated in advanced stages of post-meiosis spermatogenesis, i.e. in spermatocytes and spermatids; and their function was suggested to be involved in chromatin remodeling [120–122].

Slightly over-expressed in the Percoll-enriched cells (log_2_FC=2.7), is the Ago3 Piwi domain, a protein of the Argonaute (Ago) protein family, which is required for the self-renewal of germ-line stem cells as it maintains transposon silencing in the germ-line genome through its interaction with small Piwi-associated RNA (piRNA) [123–125]. The proliferating cell nuclear antigen (*pcna*), which was also over-expressed in the Percoll-enriched testis cells, plays an important role in BSG proliferation initiation before entering meiosis [126]. Pcna was used as a molecular marker for successful proliferation of transplanted yellowtail (*Seriola quinqueradiata*) spermatogonia after GCT into nibe croaker (*Nibea mitsukurii*) surrogate hosts [127].

#### Somatic cell-specific genes

Other genes that demonstrated over-expression in the crude testis cells are genes that are known to be expressed in somatic cells, supporting the developing germ cells: anti müllerian hormone and its receptor (*amh*/*amhr2*), *lh* and *fsh receptors*, gonadal soma-derived factor 1 (*gsdf1*), insulin growth factor 1 receptor (*igf1r*), *sox9* and *pdgf* (Fig. 8b). *Gsdf1* was shown to localize predominately in somatic cells surrounding spermatogonia in the testis of medaka (*O. latipes*) and rainbow trout (*O. mykiss*) and plays an essential role in spermatogonia differentiation [128, 129]. Expressed by Sertoli cells, platelet-derived endothelial cell growth factor (PD-ECGF) and members of the transforming growth factor (TGF-b) superfamily of glycoproteins (*activins*, *inhibins* and *amh*), mediate spermatogonia proliferation and differentiation under control of the sex steroids E2 and 11-Ketotestosterone (11KT) (reviewed in [114, 130]). The insulin-like growth factor 1 (Igf1) plays a key role in regulation of bony fish growth, differentiation and reproduction and was shown to stimulate proliferation of testicular germ cells *in vivo* [131, 132]. It appears that Igf1 can directly stimulate germ cells, thanks to the localization of its receptor (Igf1r) both in Sertoli cells and germ cells (i.e., ASG) [133, 134]. The *igf1r* was moderately over-expressed (log_2_FC=4.1) in SBT crude testis cells.

Unlike the mammalian *Sox9* gene, which is considered a male-determining gene [83], the medaka (*O. latipes*) and zebrafish (*D. rerio*) *sox9b* is not necessary for testis differentiation, but it is required for germ cell proliferation and survival. In medaka, somatic cells supporting undifferentiated germ cells, organized in units named ‘germinal cradles’, were shown to express *sox9b* predominately in males (Sertoli cells surrounding spermatogonia), but also in females (granulosa cells surrounding oogonia) [135].

#### Crude testis *vs.* Percoll-enriched testis cells DE summary

Both analyses, the GO molecular function GSEA and the specific gonad development DE genes, found consistent differential gene expression patterns between the Percoll-enriched and the crude testis cells. The genes found to be over-expressed at the Percoll-enriched testis cells, compared with crude testis cells, are known to be expressed exclusively in germ cells: *ago3 piwi domain*, *pcna*, *ipo4*, *ipo5* and *sox30*. This expression pattern, together with over-expression of somatic cell specific genes (such as the Leydig cells-expressed *3b-hsd*) in the crude testis cells, demonstrates that the Percoll enrichment method successfully enriched testicular germ cell populations over somatic cells. However, most well-known ASG-specific markers (*ly75*, *plzf*, *notch1*, *nanos1* and *gfra1*) were found to be slightly over-expressed in the crude testis cells rather than in the Percoll-enriched cells, suggesting that the Percoll-enrichment protocol failed to enrich ASG. The over-expression of *pcna*, *ipo4* and *ipo5* in the Percoll-enriched cells indicates the enrichment of differentiated testicular germ cells that are towards, or post meiosis, such as proliferating BSG and post-meiosis spermatocytes and spermatids, in the cell suspension.

The Percoll-enrichment method used in this study did not appear to yield high proportions of enriched ASG compared with crude testis cells, yet this enrichment method is known to obtain transplantable ASG, capable of migrating and colonizing the host gonads, as demonstrated both by this group [15] and in previous studies [11, 127, 136]. A single pluripotent germ cell was demonstrated to be sufficient for successful GCT and therefore even a lowly ASG-enriched cell suspension is suitable for the purpose of GCT [137]. The ASG enrichment protocol can be optimized by experimentally changing the Percoll concentration in each layer of the gradient, as previously suggested in [12, 138], as well as by dissecting just the peripheral, ASG-rich part of the testis, as demonstrated in [15]. Such improvements could achieve better enrichment of transplantable ASG, which has the potential to increase the rate of successful migration and colonization of the transplanted ASG in the surrogate host gonads [139].

### Germ cell separation methods for DE analysis

For the purpose of differential gene expression and cell type-specific gene discovery, which require better separation between germ cell populations in ovary and testis cells, more stringent cell selection methods might be required. Such methods include single-cell laser capture micro-dissection [140], or specific green fluorescence protein (GFP) labeling of the cells, followed by fluorescence activated cell sorting (FACS) [141–144]. It is worth noting though, that the use of GFP-labeled germ cells for FACS requires molecular engineering methods, which are not always applicable for non-model species lacking available gene data, such as the SBT.

### Additional annotated germ cell-specific genes

Additional germ cell-specific genes that did not pass the threshold for significantly DE genes were identified from the annotated SBT gonadal cells transcriptome. These include *vasa* and *dead-end*, both used extensively as germ cell markers in fish gonad development and GCT studies [13, 15, 145–149]. These studies have shown that *vasa* is expressed in all germ cell populations, starting from the embryonic primordial germ cells (PGC), through all levels of gametogenesis [140, 150–152], while *dead-end* was found more specific to undifferentiated germ cell populations (PGC, ASG and oogonia) [15, 107, 139]. The presence of these genes in germ cell populations in both ovaries and testes explains why they have not been detected as significantly DE.

Discovering the full sequence of these genes in SBT enabled the development of SBT-specific germ cell markers that were used successfully for both *in situ* hybridization and PCR analyses of transplanted SBT spermatogonial cells in a GCT study performed by this group [15].

## Conclusions

A high throughput analysis of gene expression in SBT gonad cells was performed in this study. The majority of genes described in this study were sequenced for the first time in SBT. The wealth of SBT gonadal and germ cell-related gene sequences identified (complete list in Additional files 2 and 4) provide a valuable resource for further reproductive molecular biology studies of the commercially important SBT and stands out in comparison to the low number of annotated tuna (*Thunnus* spp.) genes currently available. SBT ovaries and testes were found to have clearly distinct gene expression patterns, with thousands of DE genes involved in tissue structure, metabolism, cellular processes and gonad development. *In silico* enrichment of genes that are specific to, or play a major role in gonad development and germ cell molecular functions allowed for a more focused investigation of their DE patterns. The expression patterns of these gonadal genes supported the observed differences in sexual maturation stages between the sampled male and female fish and expand the existing molecular knowledge of sex differentiation and sexual maturation in SBT. Based on the differential gene expression between crude testis and Percoll gradient germ cell-enriched fraction, we suggest that the Percoll enrichment method, which is currently used in GCT experiments, should be adjusted and tested for the specific study species.

Furthermore, a shortlist of genes associated with specific germ cell activity were identified (Table 6). These may be used as SBT-specific germ cell molecular markers to distinguish between transplanted SBT germ cells and the surrogate host endogenous germ cells in future GCT studies. These genes could also be used in molecular studies of germ and soma cell interactions, in an attempt to unveil the molecular mechanisms governing germ cell proliferation and maturation to try and artificially recover these functions in naturally non-compatible GCT surrogate hosts.

**Table 6:** Southern bluefin tuna germ cell-specific genes from transcriptome assembly of gonad cells

ORF id	Length (bp)	Gene^a^	CDs coverage^b^	Annotated species	References^c^
m.177200	1104	*cxcr4*	Complete	*Stegastes partitus*	[107, 118, 119, 153, 154]
m.21888	1173	*gfra1*	Complete	*Xiphophorus maculatus*	[107, 155–158]
m.72178	1647	*ly75*	Complete	*Haplochromis burtoni*	[8, 107, 159]
m.200580	681	*nanos1*	Complete	*S. partitus*	[160–162]
m.83114	483	*nanos2*	Complete	*Dicentrarchus labrax*	[107, 156, 163, 164]
m.26953	5511	*notch1*	5’ partial	*S. partitus*	[107, 165]
m.156253	4419	*pdgfra*	Complete	*S. partitus*	[103, 116, 166]
m.166899	1434	*plzf*	3’ partial	*O. niloticus*	[107, 155]
m.3709	1431	*pou5f1/oct4*	Complete	*Larimichthys crocea*	[107, 109, 156, 167, 168]
m.15308	1284	*wt1*	Complete	*Maylandia zebra*	[115, 116]
m.155583, m.127269	1314	*dead end*	Complete	*T. orientalis*	[15, 107, 139]
m.162404	1935	*vasa*	Complete	*T. orientalis*	[140, 150–152]

^a^ORFs were annotated by the NCBI non-redundant (nr) protein database, with an *e*-value <1×10^−3^. Gene full names and details are provided in sections ‘Germ cell-specific genes’, ‘Germ cell-specific genes’ and ‘Additional annotated germ cell-specific genes’. ^b^ORF coding regions (CDs) predicted by TransDecoder from each transcript and considered as complete based on the presence of a starting methionine and an ending stop codon. ^c^Studies indicating germ cell specificity of the gene

## Additional files

Additional file 1
**Gene ontology (GO) terms enrichment in Southern bluefin tuna ovary** ***vs.*** **testis cells transcriptome.** Enriched GO terms in ovary *vs.* testis cells, along with the corresponding ontology, term description, number of genes associated (total count and differentially expressed) and the *p*-value and *p*-value (false discovery rate adjusted *p*-value) statistics as determined by goseq. (XLSX 50.5 kb)

Additional file 2
**Differentially expressed gonad development and germ cell-related genes in ovary** ***vs.*** **testis cells.** ORF – Open reading frame. Blast hit species, *e*-value, Bit Score – Blast best hit details. Log_2_FC – Fold change values (log2 transformed) as determined by edgeR, positive values represent over-expression in testis cells, negative values are over-expressed in the ovary cells. *p*-value, FDR (false discovery rate adjusted *p*-value) – edgeR differential expression analysis statistics. KEGG – Kyoto encyclopedia of genes and genomes function. (XLSX 35.7 kb)

Additional file 3
**Gene ontology (GO) terms enrichment in Southern bluefin tuna crude testis** ***vs.*** **Percoll-enriched testis cells transcriptome.** Enriched GO terms in crude testis *vs.* Percoll-enriched testis cells, along with the corresponding ontology, term description, number of genes associated (total count and differentially expressed) and the *p*-value and *p*-value (false discovery rate adjusted *p*-value) statistics as determined by goseq. (XLSX 71.6 kb)

Additional file 4
**Differentially expressed gonad development and germ cell-related genes in crude** ***vs.*** **Percoll-enriched testis cells.** ORF – Open reading frame. Blast hit species, *e*-value, Bit Score – Blast best hit details. Log_2_FC – Fold change values (log2 transformed) as determined by edgeR, positive values represent over-expression in testis cells, negative values are over-expressed in the ovary cells. *p*-value, FDR (false discovery rate adjusted *p*-value) – edgeR differential expression analysis statistics. KEGG – Kyoto encyclopedia of genes and genomes function. (XLSX 26.5 kb)
